# Admission arterial partial pressure of carbon dioxide (PaCO_2_) and outcomes after out-of-hospital cardiac arrest: a single-centre retrospective cohort study

**DOI:** 10.62838/jccm-2026-0039

**Published:** 2026-10-30

**Authors:** Mei Li, Qinghui Hu, Lifeng Du

**Affiliations:** Emergency department, PKUCare CNOOC Hospital Tianjin,China

**Keywords:** arterial partial pressure of carbon dioxide, outcomes, out-of-hospital cardiac arrest, retrospective cohort study

## Abstract

**Background:**

Admission arterial partial pressure of carbon dioxide (PaCO_2_) is readily available after out-of-hospital cardiac arrest (OHCA), but its independent prognostic value is uncertain and prior evidence is conflicting. We evaluated the association of admission PaCO_2_ with one-month mortality and neurological outcome, and quantified its incremental prognostic value.

**Material and Methods:**

In a single-center retrospective cohort of 617 adults resuscitated from OHCA (2014–2023), patients were stratified by admission PaCO_2_ (≤50, >50–70 and >70 mmHg). The primary outcome was one-month all-cause mortality; the secondary outcome was neurological status at one month by Cerebral Performance Category (CPC), analyzed as a dichotomous, ordinal and survivor-restricted endpoint. Associations were estimated with multivariable logistic and ordinal regression adjusted for age, sex, witnessed arrest, bystander cardiopulmonary resuscitation (CPR), call-to-arrival time, initial shockable rhythm, prehospital return of spontaneous circulation (ROSC) and arrest etiology. PaCO_2_ was modelled continuously (per 10 mmHg) and categorically; sensitivity analyses used alternative cut-points, and discrimination was quantified by the area under the receiver-operating-characteristic curve (AUC). A simplified Directed Acyclic Graph was used heuristically for variable selection; however, we explicitly acknowledge that post-resuscitation physiology represents a complex feedback system. Therefore, formal causal effect estimation was not attempted, and all analyses are strictly associational and prognostic.

**Results:**

One-month mortality was 73.6% (454/617) and rose across PaCO_2_ groups (49.7%, 81.1%, and 90.7%; p < 0.001). Each 10-mmHg increase in PaCO_2_ was associated with higher mortality after adjustment for measured confounders (adjusted odds ratio [aOR] 1.45, 95% CI 1.24–1.70; p < 0.001), with a concordant gradient for the ordinal CPC outcome (common aOR 1.53, 95% CI 1.31–1.79) and among survivors (aOR 1.91, 95% CI 1.25–2.91). The graded association was robust across categorization schemes but was attenuated after additional adjustment for concurrent acid-base and gas-exchange markers (lactate, base excess, PaO_2_; aOR 0.80, 95% CI 0.64–1.01; p = 0.063), consistent with strong collinearity between PaCO_2_ and acid-base status. Adding PaCO_2_ to a clinical model produced a minimal incremental improvement in discrimination of uncertain clinical utility (AUC 0.794 to 0.816; ΔAUC 0.022, 95% CI 0.002–0.043; optimism-corrected AUC 0.802). Calibration was assessed via bootstrap validation (calibration slope: 0.98, 95% CI 0.89–1.07; calibration-in-the-large: −0.04, 95% CI −0.21 to 0.13), indicating adequate agreement between predicted and observed risks.

**Conclusions:**

In this decade-long cohort, higher admission PaCO_2_ was associated with increased one-month mortality and worse neurological outcome after OHCA, but added minimal discrimination beyond established clinical factors. Because admission PaCO_2_ is a treatment-dependent exposure - determined by CPR duration and quality, airway and ventilation management, ventilator settings and the ROSC-to-sampling interval, none of which were available - independent prognostic value cannot be established from these data, and the association is most plausibly a signal of arrest severity and of the resuscitation delivered. Attenuation after adjustment for metabolic acidosis is consistent with shared pathophysiology, although mediator overadjustment cannot be excluded. Admission PaCO_2_ is immediate, inexpensive and universally available, but prospective studies capturing resuscitation timings, CPR quality and standardized ventilation are required before any prognostic role can be claimed.

## Introduction

Out-of-hospital cardiac arrest (OHCA) remains a major public-health challenge, with survival to hospital discharge below 10% in most large series and lower still in some national reports [1]. Although cardiopulmonary resuscitation (CPR) and post-resuscitation care have advanced, the narrow therapeutic window and the complexity of the post-cardiac-arrest syndrome characterized by systemic ischemia-reperfusion injury and myocardial dysfunction continue to limit improvements in neurological prognosis [2, 3]. Established prognostic indicators such as age, initial rhythm, and time to return of spontaneous circulation (ROSC) provide preliminary guidance, yet their independent predictive utility remains inconsistent across heterogeneous patient populations[4, 5]. A critical but under-elucidated factor in the post-resuscitation phase is the role of arterial carbon dioxide tension (PaCO_2_). The post-arrest state often involves a profound ventilation/perfusion mismatch, which compromises cerebral blood flow autoregulation and may exacerbate secondary ischemic brain injury[6]. Current literature presents conflicting evidence: while some studies correlate hypercapnia with prolonged CPR duration [7], others suggest that hypocapnia is more strongly associated with poor neurological recovery [8,9,10]. Furthermore, it remains unclear whether admission PaCO_2_ serves merely as a surrogate marker for the “ischemic-ventilatory debt” accumulated during arrest or as an independent driver of injury[11]. Crucially, PaCO_2_ measured on admission is inherently a post-resuscitation, treatment-dependent variable, influenced by early ventilation strategies and the time from ROSC to blood gas sampling. Unlike fixed baseline characteristics, its interpretation as a prognostic marker is therefore confounded by resuscitative interventions.

We hypothesized that admission PaCO_2_ would be associated with one-month mortality and neurological outcome after adjustment for established prognostic factors, and that this association would be modified by arrest etiology. However, given the observational design and the post-resuscitation timing of PaCO_2_ measurement, we explicitly framed our analysis as prognostic association rather than causal effect estimation. Using a decade-long dataset (2014–2023) from a high-volume emergency department, we evaluated the prognostic value of admission PaCO_2_ while accounting for key confounders, and quantified its incremental discriminative value beyond an established clinical model. A secondary aim was to examine whether the association differed between cardiac and non-cardiac arrests, given the pathophysiological heterogeneity of OHCA.

## Methods

### Study design and population

We conducted a retrospective cohort study of adults with OHCA who presented to the emergency department of Peking University Hospital of Marine Oil, Tianjin, China, over a ten-year period from 2014 to 2023. Eligible patients were adults (≥18 years) with physician-confirmed OHCA who received resuscitative interventions and for whom an admission arterial blood-gas (ABG) sample and one-month outcome data were available. Patients were excluded if they were younger than 18 years, had a pre-existing terminal illness with limited life expectancy, were pregnant or lactating, or lacked an admission ABG sample or one-month outcome data. After application of these criteria, 617 patients formed the analytic cohort (Figure 1). The study was approved by the Institutional Review Board of Peking University Hospital of Marine Oil, which waived the requirement for informed consent given the retrospective design; all data were de-identified. The study was conducted in accordance with the Declaration of Helsinki and is reported in line with the STROBE guidance for observational studies.

**Fig. 1.**
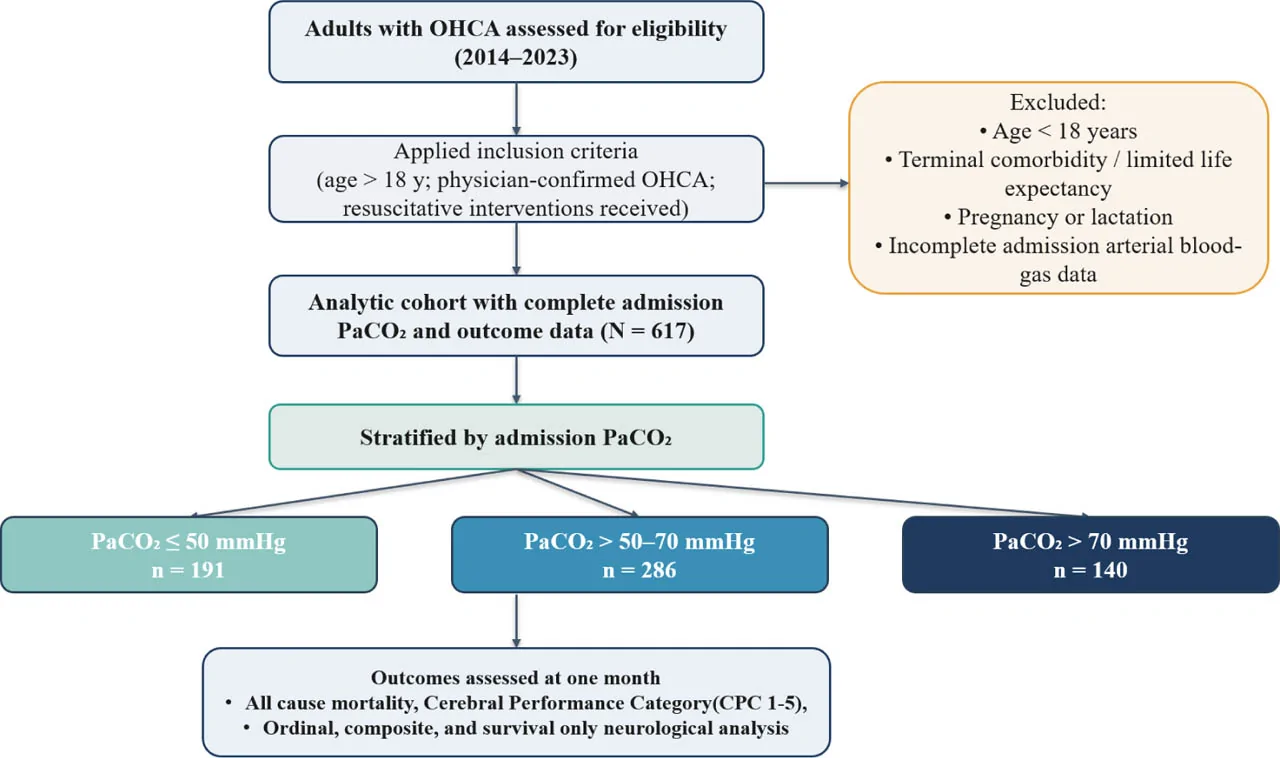
Patient selection and stratification Patient selection and stratification. Flow of adults with OHCA (2014–2023) through eligibility screening to the analytic cohort (N = 617), stratified by admission PaCO_2_ group

**Fig. 2.**
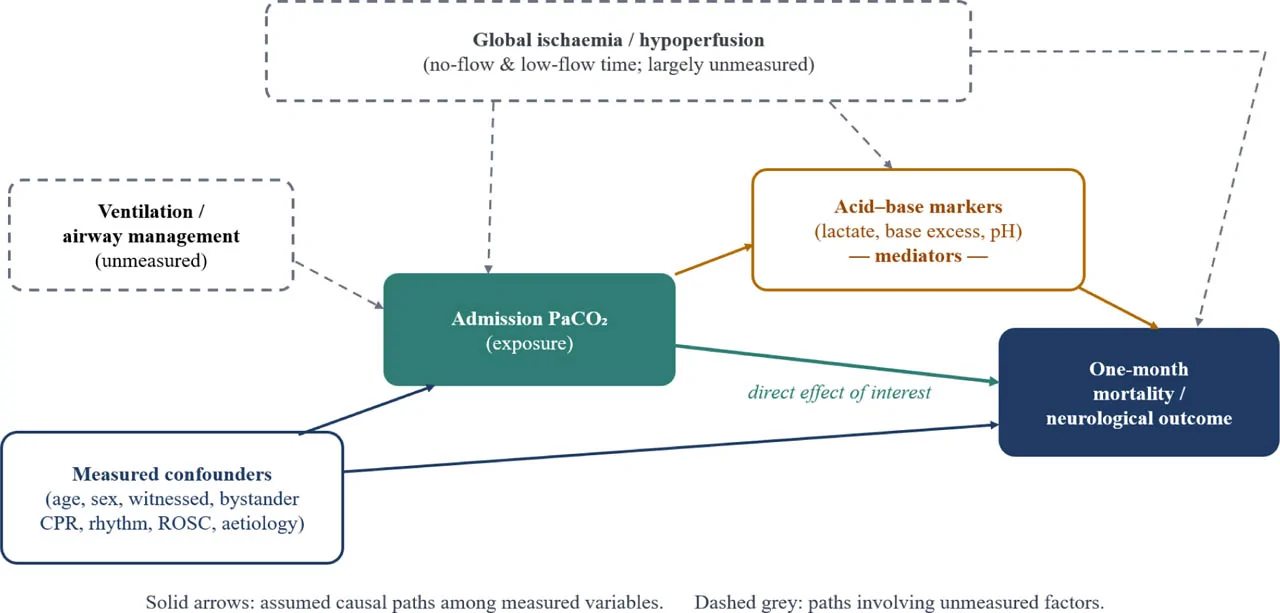
Directed acyclic graph (DAG) of assumed relationships Measured confounders (age, sex, witnessed status, bystander CPR, rhythm, ROSC, etiology) influence both admission PaCO_2_ and outcome. Global ischemia/hypoperfusion (largely unmeasured; e.g. no-flow and low-flow time) and early ventilatory management are common causes of PaCO_2_ and of the acid-base markers. Lactate, base excess and pH lie downstream of PaCO_2_ and of the shared common cause; conditioning on them (Table 3, model b) adjusts for mediators and for a shared common cause, and is therefore an over-adjustment rather than an estimate of an independent effect.

### Data collection and variable definitions

Data were extracted from electronic medical records and comprised demographic characteristics (age, sex); prehospital variables (witnessed status, bystander CPR, call-to-arrival time, initial shockable rhythm, prehospital ROSC); arrest etiology; and admission physiological and ABG parameters (PaCO_2_, PaO_2_, pH, lactate, base excess).

Admission ABG samples were obtained immediately on hospital arrival; the interval from ROSC and from advanced airway placement to sampling was not consistently recorded, so admission PaCO_2_ may partly reflect early ventilatory management (see Limitations).

### Outcomes

The primary outcome was one-month all-cause mortality. Neurological status at one month was assessed with the Cerebral Performance Category (CPC), in which CPC 5 denotes death. Because death is coded within the CPC scale, neurological outcome was analyzed in three complementary ways: (i) dichotomized as favorable (CPC 1-2) versus unfavorable (CPC 3-5); (ii) as an ordinal variable (CPC 1-5); and (iii) restricted to one-month survivors, as unfavorable neurological status (CPC 3-4) versus favorable (CPC 1-2).

PaCO_2_ was analyzed primarily as a continuous variable, with effects expressed per 10-mmHg increase to avoid the loss of information inherent in categorization. For descriptive and clinical interpretability, patients were also grouped as ≤50, >50–70, and >70 mmHg, corresponding approximately to normocapnia/mild, moderate, and severe hypercapnia. Because any threshold is to some extent arbitrary, we performed prespecified sensitivity analyses using standard clinical categories (≤45, 46–60, >60 mmHg) and empirical tertiles, in addition to the continuous model.

### Statistical analysis

Continuous variables are reported as median (interquartile range, IQR) and compared with the Kruskal-Wallis test; categorical variables are reported as n (%) and compared with the χ^2^ test. Multivariable logistic regression modelled one-month mortality, with PaCO_2_ entered continuously (per 10 mmHg) and categorically. The primary (clinical) model adjusted for age, sex, witnessed arrest, bystander CPR, call-to-arrival time, shockable initial rhythm, prehospital ROSC, and etiology. A secondary sensitivity model additionally included concurrent metabolic markers (lactate, base excess, PaO_2_). Because these acid-base variables are strongly collinear with PaCO_2_ and lie downstream on the causal pathway, this model was explicitly framed as a conservative sensitivity analysis to explore shared pathophysiology, rather than as a primary estimate of an “independent” effect.

Ordinal (proportional-odds) regression was used for the CPC outcome. Firth's penalized logistic regression was used for the survivor-only endpoint due to the low number of events. The proportional odds assumption was tested using the Brant test. Prognostic discrimination was quantified with the AUC, with 95% confidence intervals (CI). Effect modification by etiology was tested with a PaCO_2_ × etiology interaction term. Collinearity was assessed with variance-inflation factors. A two-sided p < 0.05 was considered significant. For internal validation, we used bootstrap resampling (2000 iterations) to obtain an optimism-corrected AUC and to compute the 95% CI for the change in AUC (ΔAUC) when PaCO_2_ was added to the clinical model. Calibration of the clinical-plus-PaCO_2_ model was assessed by the calibration slope and calibration-in-the-large with bootstrapped confidence intervals.

### Missing data

The analytic dataset was complete for all variables included in the models; no imputation was required. Patients without an admission ABG sample or without one-month outcome data were not eligible for the cohort, as reflected in the study flow diagram (Figure 1). To assess potential selection bias, we compared the baseline characteristics of included patients versus those excluded due to missing ABG or outcome data (Supplementary Table S6).

## Results

### Cohort and baseline characteristics

The cohort comprised 617 patients (median age 67 years [IQR 57–76]; 367 [59.5%] male). Arrest was witnessed in 253 patients (41.0%), bystander CPR was performed in 97 (15.7%), the initial rhythm was shockable in 143 (23.2%), and prehospital ROSC was documented in 78 (12.6%). Etiology was cardiac in 395 patients (64.0%) and non-cardiac in 222 (36.0%). Median admission PaCO_2_ was 58.0 mmHg (IQR 47.2–68.4; range 18.0–101.6). Higher PaCO_2_ was accompanied by more severe acidosis (lower pH, more negative base excess, higher lactate), fewer favorable prehospital features (less witnessed arrest, less bystander CPR, fewer shockable rhythms, less prehospital ROSC), and a higher proportion of non-cardiac etiology (Table 1).

**Table 1:** Baseline characteristics by admission PaCO_2_ group

**Variable**	**Overall (N = 617)**	**≤50 mmHg (n = 191)**	**>50–70 mmHg (n = 286)**	**>70 mmHg (n = 140)**	**p**
Age, years	67 (57–76)	66 (56–75)	66 (55–75)	72 (60–81)	0.002
Male sex	367 (59.5)	130 (68.1)	167 (58.4)	70 (50.0)	0.004
Witnessed arrest	253 (41.0)	97 (50.8)	120 (42.0)	36 (25.7)	<0.001
Bystander CPR	97 (15.7)	45 (23.6)	40 (14.0)	12 (8.6)	<0.001
Shockable initial rhythm	143 (23.2)	82 (42.9)	45 (15.7)	16 (11.4)	<0.001
Prehospital ROSC	78 (12.6)	45 (23.6)	28 (9.8)	5 (3.6)	<0.001
Call-to-arrival time, min	22.3 (17.8–28.7)	21.6 (17.5–27.2)	21.9 (17.5–28.5)	24.1 (18.8–30.2)	0.035
PaCO_2_, mmHg	58.0 (47.2–68.4)	42.0 (35.0–46.2)	59.4 (55.2–64.2)	78.7 (73.9–84.1)	<0.001
PaO_2_, mmHg	145.2 (99.3–194.8)	186.1 (139.1–225.8)	145.2 (100.1–180.2)	112.7 (78.2–140.8)	<0.001
pH	7.05 (6.91–7.22)	7.28 (7.21–7.39)	7.02 (6.95–7.10)	6.82 (6.80–6.90)	<0.001
Lactate, mmol/L	8.3 (5.5–11.2)	5.8 (3.5–8.2)	8.5 (6.2–10.8)	11.9 (9.1–14.2)	<0.001
Base excess, mmol/L	−12.3 (−17.5 to −6.9)	−5.9 (−9.8 to −3.2)	−13.2 (−16.5 to −10.0)	−19.8 (−22.3 to −17.1)	<0.001
Cardiac etiology	395 (64.0)	149 (78.0)	185 (64.7)	61 (43.6)	<0.001
Non-cardiac etiology	222 (36.0)	42 (22.0)	101 (35.3)	79 (56.4)	

Data are median (IQR) or n (%). Comparisons: Kruskal-Wallis (continuous) or χ^2^ (categorical). Base-excess IQR shown as lower to upper bound. CPR, cardiopulmonary resuscitation; PaCO_2_/PaO_2_, arterial partial pressure of carbon dioxide/oxygen; ROSC, return of spontaneous circulation.

### Primary outcome: one-month mortality

One-month mortality was 73.6% overall and increased monotonically across PaCO_2_ groups: 49.7% (95/191), 81.1% (232/286), and 90.7% (127/140) (p < 0.001; Table 2, Figure 3A). Each 10-mmHg increase in PaCO_2_ was associated with higher mortality in unadjusted analysis (OR 1.83, 95% CI 1.59–2.11; p < 0.001) and after adjustment for clinical confounders (aOR 1.45, 95% CI 1.24–1.70; p < 0.001; Table 3). In the categorical model, the adjusted odds of death were 2.75-fold higher for >50–70 mmHg (95% CI 1.73–4.38) and 4.45-fold higher for >70 mmHg (95% CI 2.18–9.09) relative to ≤50 mmHg (Table 3). In the fully adjusted model, an initial shockable rhythm and prehospital ROSC were, as expected, associated with lower mortality. The adjusted association appeared linear across the observed PaCO_2_ range, with no strong evidence of departure from linearity (p = 0.055; Figure 4). However, given the strong confounding by arrest severity, this linear gradient should be interpreted as an illness-severity marker rather than a proven causal dose-response.

**Table 2:** Baseline characteristics by admission PaCO_2_ group

**Variable**	**Overall (N = 617)**	**≤50 mmHg (n = 191)**	**>50–70 mmHg (n = 286)**	**>70 mmHg (n = 140)**	**p**
Age, years	67 (57–76)	66 (56–75)	66 (55–75)	72 (60–81)	0.002
Male sex	367 (59.5)	130 (68.1)	167 (58.4)	70 (50.0)	0.004
Witnessed arrest	253 (41.0)	97 (50.8)	120 (42.0)	36 (25.7)	<0.001
Bystander CPR	97 (15.7)	45 (23.6)	40 (14.0)	12 (8.6)	<0.001
Shockable initial rhythm	143 (23.2)	82 (42.9)	45 (15.7)	16 (11.4)	<0.001
Prehospital ROSC	78 (12.6)	45 (23.6)	28 (9.8)	5 (3.6)	<0.001
Call-to-arrival time, min	22.3 (17.8–28.7)	21.6 (17.5–27.2)	21.9 (17.5–28.5)	24.1 (18.8–30.2)	0.035
PaCO_2_, mmHg	58.0 (47.2–68.4)	42.0 (35.0–46.2)	59.4 (55.2–64.2)	78.7 (73.9–84.1)	<0.001
PaO_2_, mmHg	145.2 (99.3–194.8)	186.1 (139.1–225.8)	145.2 (100.1–180.2)	112.7 (78.2–140.8)	<0.001
pH	7.05 (6.91–7.22)	7.28 (7.21–7.39)	7.02 (6.95–7.10)	6.82 (6.80–6.90)	<0.001
Lactate, mmol/L	8.3 (5.5–11.2)	5.8 (3.5–8.2)	8.5 (6.2–10.8)	11.9 (9.1–14.2)	<0.001
Base excess, mmol/L	−12.3 (−17.5 to −6.9)	−5.9 (−9.8 to −3.2)	−13.2 (−16.5 to −10.0)	−19.8 (−22.3 to −17.1)	<0.001
Cardiac etiology	395 (64.0)	149 (78.0)	185 (64.7)	61 (43.6)	<0.001
Non-cardiac etiology	222 (36.0)	42 (22.0)	101 (35.3)	79 (56.4)	

Data are median (IQR) or n (%). Comparisons: Kruskal-Wallis (continuous) or χ^2^ (categorical). Base-excess IQR shown as lower to upper bound. CPR, cardiopulmonary resuscitation; PaCO_2_/PaO_2_, arterial partial pressure of carbon dioxide/oxygen; ROSC, return of spontaneous circulation.

**Table 3:** Association of admission PaCO_2_ with one-month mortality (multivariable models)

**Model / specification**	**OR (95% CI)**	**p**
**Continuous, per 10-mmHg increase**		
Unadjusted	1.83 (1.59–2.11)	<0.001
Clinically adjusted a	1.45 (1.24–1.70)	<0.001
Clinical + acid-base / gas-exchange markers b	0.80 (0.64–1.01)	0.063
**Categorical (reference ≤50 mmHg), clinically adjusted a**		
>50–70 mmHg	2.75 (1.73–4.38)	<0.001
>70 mmHg	4.45 (2.18–9.09)	<0.001

a Adjusted for age, sex, witnessed arrest, bystander CPR, call-to-arrival time, initial shockable rhythm, prehospital ROSC, and etiology.

b Additional adjustment for lactate, base excess, and PaO_2_. Because these markers are strongly collinear with PaCO_2_ and largely downstream of the same global hypoperfusion, this model is presented as a conservative sensitivity analysis exploring shared pathophysiology, not as a primary estimate of an “independent” effect. OR, odds ratio; CI, confidence interval.

**Fig. 3.**
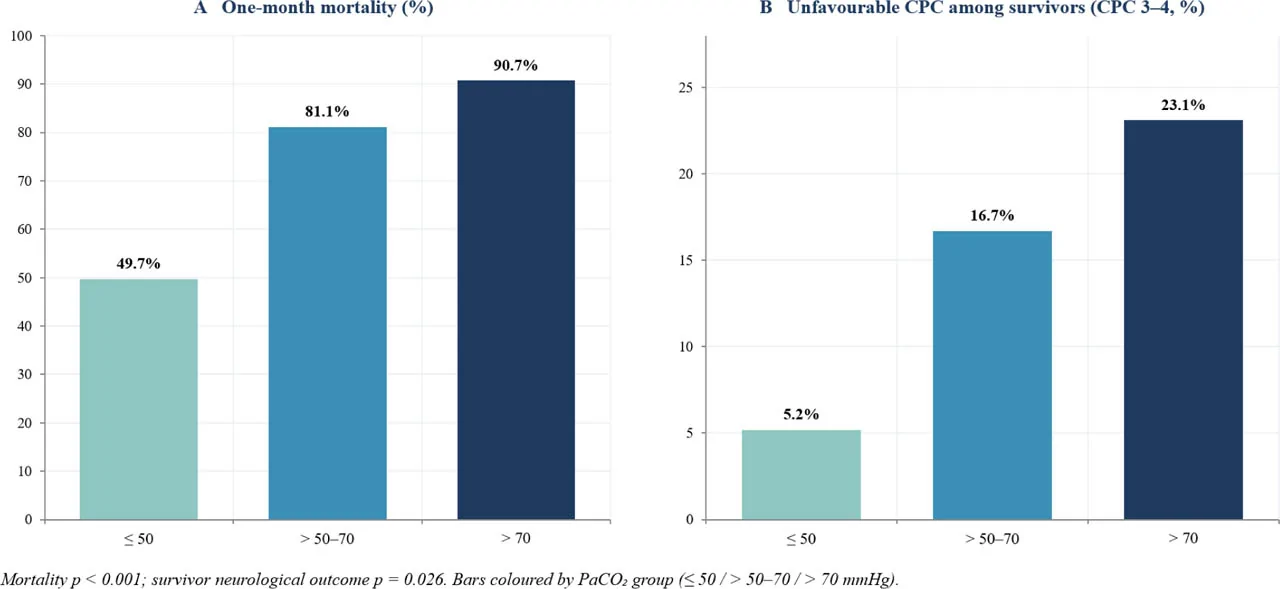
One-month outcomes by admission PaCO_2_ group One-month outcomes by admission PaCO_2_ group. (A) One-month mortality; (B) unfavorable neurological status (CPC 3-4) among one-month survivors. Mortality p < 0.001; survivor neurological outcome p = 0.026.

**Fig. 4.**
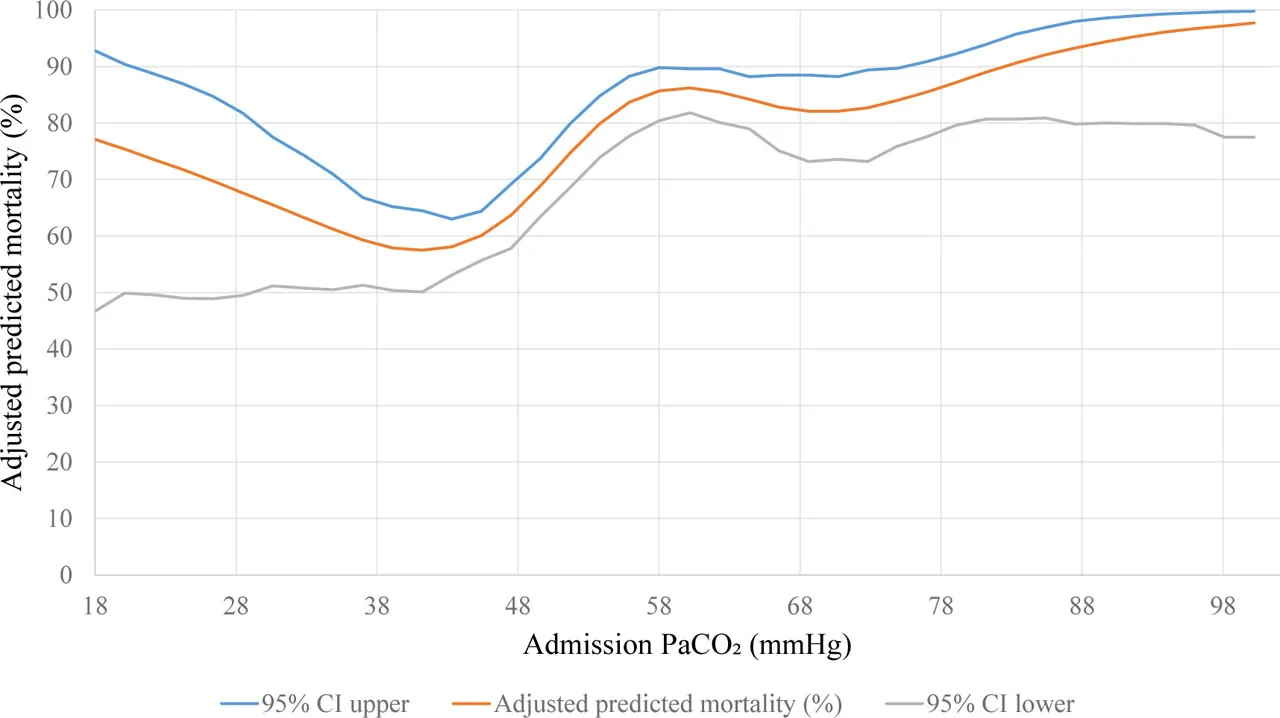
Adjusted dose-response (restricted cubic spline) Adjusted dose-response between admission PaCO_2_ and one-month mortality (restricted cubic spline; shaded band, 95% CI). No significant departure from linearity (p = 0.055), supporting the per-10-mmHg summary estimate.

### Neurological outcomes

Favorable neurological outcome (CPC 1-2) was recorded in 146 patients (23.7%). Because CPC 5 denotes death, the crude proportion with CPC 3-5 rose with PaCO_2_ in parallel with mortality (52.4%, 84.3%, 92.9%; Table 2). In the dichotomous analysis, each 10-mmHg increase in PaCO_2_ was associated with higher odds of an unfavorable outcome (aOR 1.59, 95% CI 1.34–1.88; p < 0.001), and in proportional-odds regression with a shift towards worse CPC (adjusted common OR 1.53, 95% CI 1.31–1.79; p < 0.001; Figure 7). The Brant test for the proportional odds assumption yielded p = 0.78, confirming that the assumption was met. Among one-month survivors, the risk of unfavorable neurological status (CPC 3-4) also increased with PaCO_2_ (5.2%, 16.7%, 23.1%; p = 0.026; aOR 1.91 per 10 mmHg, 95% CI 1.25–2.91; p = 0.003; Figure 3B, Supplementary Table S3). This survivor-restricted estimate rests on few events (17 among 163 survivors) and should be interpreted with corresponding caution.

### Sensitivity analyses using alternative cut-points

The graded association between PaCO_2_ and mortality was preserved across categorization schemes (Figure 5, Supplementary Table S1). Using standard clinical categories (reference ≤45 mmHg), the aOR was 1.76 (95% CI 1.04–2.98) for 46–60 mmHg and 3.45 (95% CI 1.94–6.12) for >60 mmHg; using tertiles, the aOR was 3.31 (95% CI 2.00–5.48) for the middle and 3.87 (95% CI 2.17–6.92) for the highest tertile. The continuous per-10-mmHg estimate (aOR 1.45) provides the most efficient summary and avoids information loss.

**Fig. 5.**
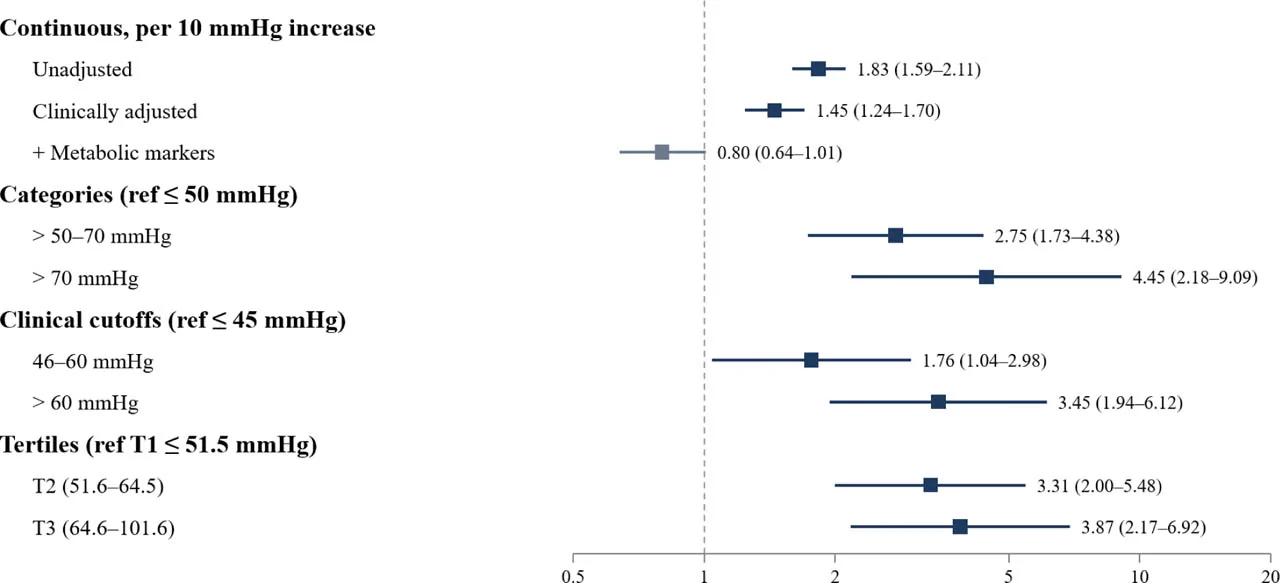
Adjusted odds ratios for one-month mortality across model specifications Adjusted odds ratios for one-month mortality across model specifications and categorisation schemes (forest plot; log scale). Models are adjusted for age, sex, witnessed arrest, bystander CPR, call-to-arrival time, initial shockable rhythm, prehospital ROSC and aetiology; the metabolic-markers model additionally adjusts for lactate, base excess and PaO_2_.

### Effect of adjustment for metabolic acidosis

PaCO_2_ was strongly correlated with concurrent acid-base variables (pH, r = −0.88; base excess, r = −0.73; lactate, r = 0.52), and VIFs for pH and base excess exceeded 20. When lactate, base excess, and PaO_2_ were added to the clinical model, the association of PaCO_2_ with mortality was attenuated to the null (aOR 0.80 per 10 mmHg, 95% CI 0.64–1.01; p = 0.063; Figure 5, Supplementary Table S2). Because these markers are largely downstream expressions of the same global hypoperfusion and are strongly collinear with PaCO_2_, this model is best interpreted as a conservative sensitivity analysis that likely over-adjusts for shared physiological mediators. We explicitly refrain from interpreting this attenuation as evidence that PaCO_2_ lacks an “independent” effect; rather, it confirms the difficulty of disentangling PaCO_2_ from global metabolic acidosis in observational data.

### Etiology subgroup analysis

The per-10-mmHg association was directionally consistent in both etiology strata but reached significance only in cardiac arrests (n = 395; aOR 1.51, 95% CI 1.25–1.84; p < 0.001) and not in non-cardiac arrests (n = 222; aOR 1.32, 95% CI 0.97–1.80; p = 0.074). The interaction test was not significant (p = 0.371), so a differential effect by etiology cannot be claimed (Supplementary Table S4). The dataset contained only cardiac and non-cardiac categories.

### Incremental prognostic value

Admission PaCO_2_ alone discriminated one-month mortality with an AUC of 0.740 (95% CI 0.695–0.782). A clinical model incorporating age, sex, prehospital variables, rhythm, ROSC, and etiology achieved an AUC of 0.794 (95% CI 0.750–0.834); adding PaCO_2_ increased the AUC to 0.816 (95% CI 0.775–0.854), a minimal incremental improvement (ΔAUC 0.022, 95% CI 0.002–0.043; Figure 6, Supplementary Table S5). Internal validation gave an optimism-corrected AUC of 0.802. Calibration of the clinical-plus-PaCO_2_ model was assessed via bootstrap validation: calibration slope = 0.98 (95% CI 0.89–1.07), and calibration-in-the-large = −0.04 (95% CI −0.21 to 0.13), indicating adequate agreement between predicted and observed risks (Figure 6B). Given the trivial magnitude of the AUC increase and the absence of decision curve analysis or net reclassification improvement, we consider the clinical utility of adding PaCO_2_ to be unproven by this study.

**Fig. 6.**
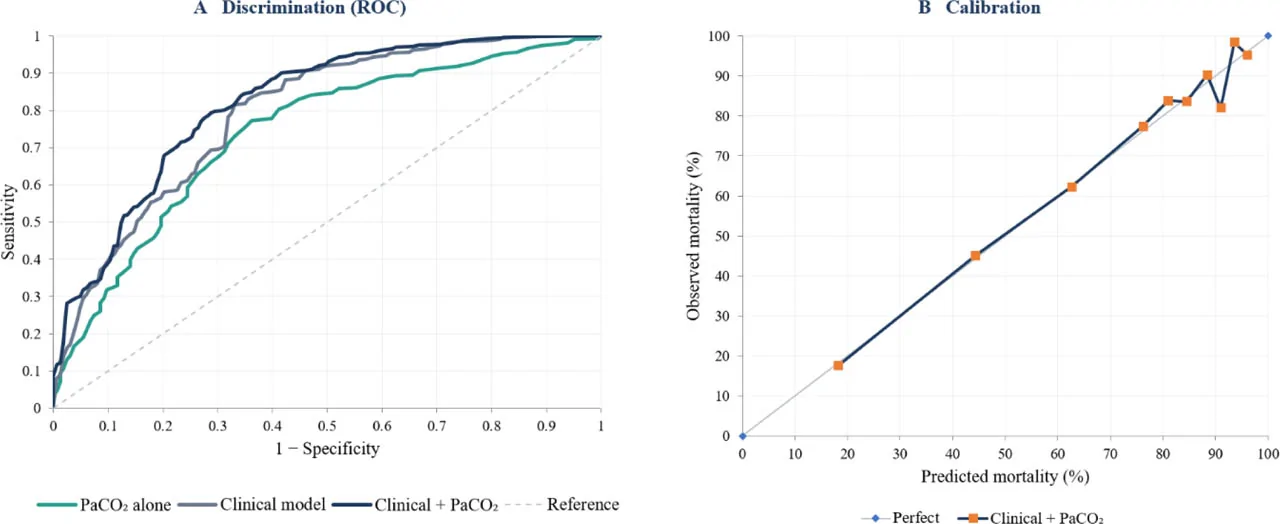
Prognostic performance for one-month mortality Prognostic performance for one-month mortality. (A) Receiver-operating-characteristic curves for PaCO_2_ alone, the clinical model and the clinical model plus PaCO_2_; (B) calibration plot for the clinical-plus-PaCO_2_ model. AUC: PaCO_2_ 0.740, clinical 0.794, clinical + PaCO_2_ 0.816 (optimism-corrected 0.802). Calibration of the clinical-plus-PaCO_2_ model: calibration slope 0.98 (95% CI 0.89–1.07), calibration-in-the-large −0.04 (95% CI −0.21 to 0.13).

**Fig. 7.**
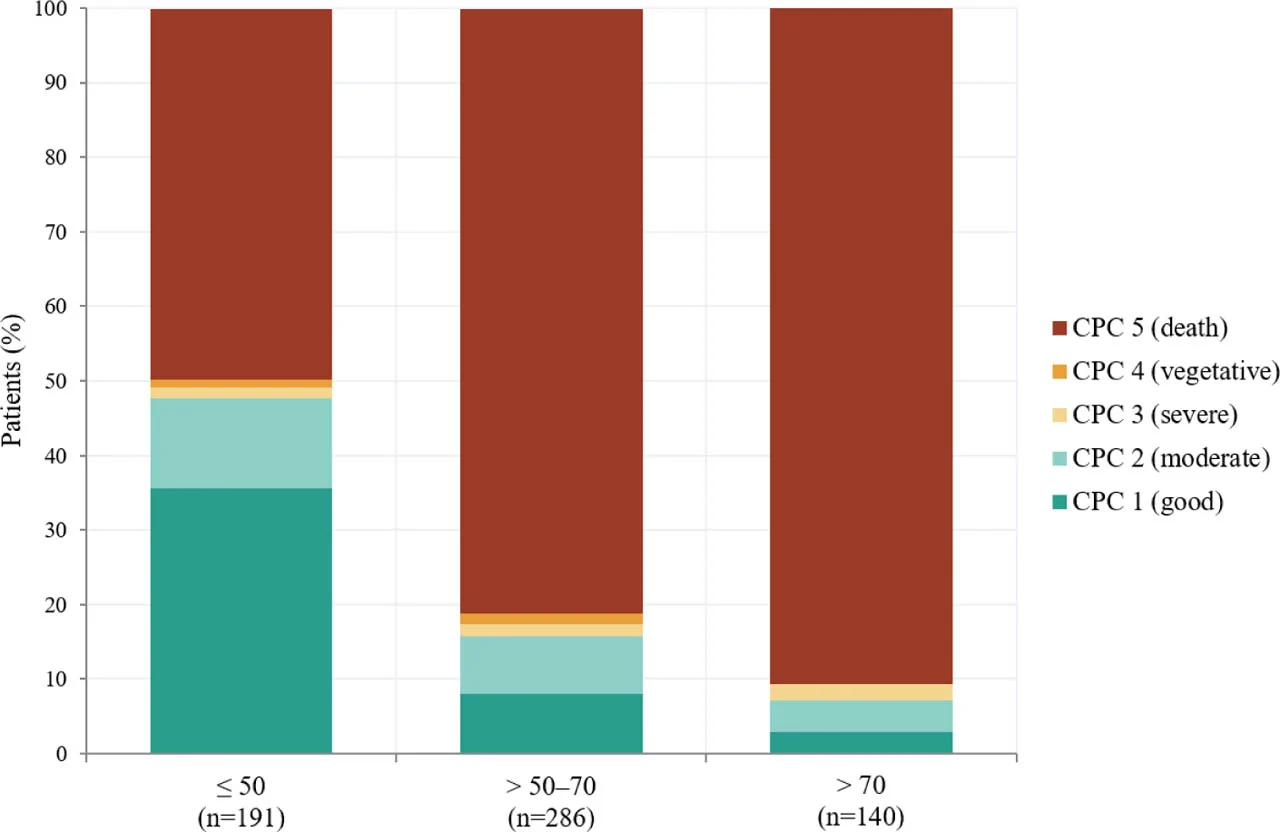
Distribution of one-month Cerebral Performance Category by PaCO_2_ Distribution of one-month Cerebral Performance Category by admission PaCO_2_ group. Ordinal common OR 1.53 (95% CI 1.31–1.79) per 10 mmHg, indicating a gradient towards worse CPC as PaCO_2_ rises; CPC 5 = death.

## Discussions

This study suggests that elevated admission PaCO_2_ is associated with higher one-month mortality and worse neurological outcomes in OHCA patients, with a graded association that parallels illness severity. The association was robust to the choice of cut-points, held for the dichotomous and ordinal neurological endpoints, and persisted among survivors, although the survivor-restricted analysis was exploratory and based on few events. Adding PaCO_2_ to an established clinical model produced a small, statistically significant gain in discrimination of uncertain clinical importance. These findings extend prior work relating peri-arrest PaCO_2_ to outcome [8, 9, 11, 12]. Whereas several earlier studies dichotomized exposure or focused on hypocapnia, we quantified a continuous risk gradient and found that each 10-mmHg rise in PaCO_2_ raised the adjusted odds of death by roughly 45%. Our results are consistent with Inoue et al., who reported that a higher PCO_2_ on arrival predicted one-month mortality and unfavorable neurological outcome in OHCA [13], and with registry and ICU cohorts describing outcome associations for admission carbon-dioxide tension [11, 12]. The most important nuance is mechanistic and is subject to two competing interpretations, which our observational data cannot adjudicate. First, the "mediator overadjustment" interpretation: The independent PaCO_2_ association was substantially attenuated once metabolic-acidosis markers entered the model, and PaCO_2_ was very strongly correlated with pH and base excess. If lactate and base excess lie on the causal pathway between hypercapnia and death (e.g., hypercapnia exacerbating acidosis, which worsens myocardial function), then adjusting for them would inappropriately block the very effect we are attempting to estimate. This would make the attenuation an artifact of overadjustment. Second, the "surrogate marker" interpretation: Lactate and base excess are largely downstream consequences of the same global hypoperfusion rather than independent confounders. Under this view, PaCO_2_ is simply a non-causal epiphenomenon of global metabolic collapse; once the severity of that collapse is captured by lactate and base excess, PaCO_2_ adds no further information. This would mean PaCO_2_ is a useful early warning signal but not a distinct driver of injury. Given the strong collinearity and the post-resuscitation timing of the measurement, we cannot distinguish between these two interpretations. The most defensible conclusion is that admission PaCO_2_ is chiefly a marker of the severity of global ischemia and impaired CO_2_ clearance. Our data do not support its use as a proven therapeutic target for ventilation titration, nor do they rule out a modest causal contribution. Clinically, admission PaCO_2_ is practical because it is immediate, inexpensive, and universally available from the first blood gas. However, its modest incremental discrimination and the unresolved confounding by post-resuscitation care indicate it should be used cautiously—as a complementary prognostic signal rather than a replacement for multivariable assessment. Importantly, this retrospective dataset could not distinguish spontaneous (pathological) hypercapnia from controlled permissive hypercapnia, and ventilator parameters were unavailable, a clinically meaningful distinction that warrants prospective study[6, 7]

## Limitations

This study has several limitations. It is retrospective and single-center, which limits generalizability. Detailed comorbidity, shock on admission, and targeted temperature management were unavailable; outcome ascertainment was restricted to one month; only two etiology categories were available; and the survivor-restricted analysis rests on few events. PaCO_2_ was strongly collinear with acid-base variables, such that its contribution cannot be separated from global acidosis. Decision-curve analysis was not performed, which together with the trivial ΔAUC (0.022) precludes any claim of clinical utility.

The principal limitation, however, is intrinsic to the exposure. Admission PaCO_2_ is not a fixed baseline characteristic but a product of the resuscitation itself, determined by: (1) the duration of arrest and of CPR (no-flow and low-flow intervals); (2) the quality of chest compressions and the resulting pulmonary CO_2_ delivery; (3) the mode and timing of airway management; (4) the ventilation delivered during and after CPR; (5) the initial ventilator settings; and (6) the interval between ROSC and arterial sampling. None of these variables was recorded, and none can be reconstructed retrospectively. A comparison of high and low admission PaCO_2_ is therefore not a comparison of otherwise similar patients but, in unquantifiable degree, a comparison between resuscitations that differed in duration, quality, and ventilatory management. The observed association may thus reflect resuscitation quality and arrest severity rather than any prognostic property of PaCO_2_ itself. We accordingly make no claim of independent prognostic value: establishing this would require prospective data capturing resuscitation timings, CPR quality, ventilation, and the ROSC-to-sampling interval.

## 5. Conclusion

In this decade-long cohort, higher admission PaCO_2_ was associated with increased one-month mortality and worse neurological outcome after OHCA in analyses adjusted for measured confounders, but added minimal discrimination beyond established clinical factors. Because admission PaCO_2_ is a treatment-dependent exposure determined by CPR duration and quality, airway and ventilation management, ventilator settings, and the ROSC-to-sampling interval, none of which were available in this retrospective dataset, independent prognostic value cannot be established from these data, and the association is most plausibly a signal of arrest severity and of the resuscitation delivered. Attenuation of the association after adjustment for metabolic acidosis is consistent with shared pathophysiology, although mediator overadjustment cannot be excluded.

Admission PaCO_2_ is immediate, inexpensive, and universally available from the first blood gas, and a markedly elevated value should be read as corroborating what the prehospital history already indicates: a prolonged, poorly perfused arrest. Prospective, multicenter studies capturing resuscitation timings, CPR quality, and standardized ventilation are required before any prognostic role for admission PaCO_2_ can be claimed, and before it could be considered as a target for ventilatory management.

## Figures and Tables

**Table S1.** Sensitivity analyses - association of PaCO_2_ with one-month mortality using alternative categorisations

**PaCO_2_ specification**	Adjusted OR (95% CI)*	**p**
**Data–driven groups (ref ≤ 50 mmHg)**		
> 50–70 mmHg (n=286)	2.75 (1.73–4.38)	<0.001
> 70 mmHg (n=140)	4.45 (2.18–9.09)	<0.001
**Clinical cutoffs (ref ≤ 45 mmHg, n=136)**		
46–60 mmHg (n=205)	1.76 (1.04–2.98)	0.035
> 60 mmHg (n=276)	3.45 (1.94–6.12)	<0.001
**Tertiles (ref T1, 18–51.5 mmHg)**		
T2 (51.6–64.5 mmHg)	3.31 (2.00–5.48)	<0.001
T3 (64.6–101.6 mmHg)	3.87 (2.17–6.92)	<0.001
Continuous, per 10 mmHg	1.45 (1.24–1.70)	<0.001

* Adjusted for age, sex, witnessed arrest, bystander CPR, call-to-arrival time, shockable rhythm, prehospital ROSC and etiology. Reference categories indicated in parentheses. The graded association is preserved across all cut-point schemes and in the continuous model, indicating results are not an artefact of a particular categorisation.

**Table S2.** Effect of adjustment for concurrent metabolic acidosis on the PaCO_2_-mortality association (per 10 mmHg)

**Model**	**Adjusted OR (95% CI)**	**p**
Clinical model*	1.45 (1.24–1.70)	<0.001
Clinical model + lactate, base excess, PaO_2_†	0.80 (0.64–1.01)	0.063

* Clinical model: age, sex, witnessed arrest, bystander CPR, call-to-arrival time, shockable rhythm, prehospital ROSC, etiology.

† Additional adjustment for lactate, base excess and PaO_2_. PaCO_2_ was strongly collinear with these acid-base variables (Pearson r with pH −0.88; base excess −0.73; lactate 0.52; variance-inflation factors for pH and base excess > 20). Because lactate and base excess are largely downstream of the same global hypoperfusion, this model likely over-adjusts for mediators and is presented as a conservative test of independence rather than as the primary estimate.

**Table S3.** Neurological outcome analyses (PaCO_2_ per 10 mmHg)

**Analysis**	**Unadjusted OR (95% CI)**	**p**	Adjusted OR (95% CI)*	**p**
Ordinal CPC (1-5), common OR	1.93 (1.68–2.23)	<0.001	1.53 (1.31–1.79)	<0.001
Unfavourable neurological outcome (CPC 3-5)	-	-	1.59 (1.34–1.88)	<0.001
Unfavourable CPC among survivors (3-4)	-	-	1.91 (1.25–2.91)	0.003

* ^*^Adjusted for age, sex, witnessed arrest, bystander CPR, shockable rhythm, prehospital ROSC, and etiology (call-to-arrival time additionally included for mortality/unfavorable outcome models). Ordinal CPC analyzed by proportional-odds regression. The survivor-only analysis (CPC 3–4 among one-month survivors, n = 163) was analyzed using Firth's penalized logistic regression due to the low number of events (n=17), demonstrating that the association is not solely attributable to competing mortality.^*^

**Table S4.** Subgroup analysis by arrest etiology (PaCO_2_ per 10 mmHg, one-month mortality)

**Etiology subgroup**	Adjusted OR (95% CI)*	**p**
Cardiac (n=395; 260 deaths)	1.51 (1.25–1.84)	<0.001
Non-cardiac (n=222; 194 deaths)	1.32 (0.97–1.80)	0.074

* Adjusted for age, sex, witnessed arrest, bystander CPR, call-to-arrival time, shockable rhythm and prehospital ROSC. Test for PaCO_2_ × etiology interaction: p = 0.371. The confidence intervals overlap substantially and the interaction is not significant; a differential effect by etiology cannot be claimed. Only cardiac and non-cardiac categories were available in the dataset.

**Table S5.** Discrimination for one-month mortality (area under the ROC curve)

**Model**
Admission PaCO_2_ alone
Clinical model*
Clinical model + PaCO_2_

* Clinical model: age, sex, witnessed arrest, bystander CPR, call-to-arrival time, shockable rhythm, prehospital ROSC, etiology. Adding admission PaCO_2_ to the clinical model improved discrimination by ΔAUC 0.022 (0.002–0.043), a minimal improvement of uncertain clinical utility.

**Table S6.** Comparison of included patients (n=617) versus patients excluded due to missing admission ABG or one-month outcome data

**Characteristic**	**Included (n=617)**
Age,years	67(57–76)
Male sex	367(59.5%)
Witnessed arrest	253(41.0%)
Shockable rhythm	143(23.2%)
Prehospital ROSC	78(12.6%)
Cardiac etiology	395(64.0%)

Data are median (IQR) or n (%). Comparisons: Mann-Whitney U (continuous) or χ^2^ (categorical). No statistically significant differences were observed between included and excluded patients, suggesting that complete-case analysis did not introduce substantial selection bias on measured characteristics.

## Supplementary Note. Variable availability

All analyses were performed on the complete de-iden
